# Aufsuchendes dyadisches Unterstützungsprogramm für Personen mit Demenz und Angehörige durch Pflegeexpert:innen Demenz: qualitative Ergebnisse einer Pilotstudie im ländlichen Raum

**DOI:** 10.1007/s00391-022-02106-7

**Published:** 2022-09-16

**Authors:** Esther Berkemer, Marion Bär

**Affiliations:** 1 Fachbereich Sozial- und Gesundheitswesen, Hochschule für Wirtschaft und Gesellschaft, Ernst-Boehe-Str. 4, 67059 Ludwigshafen am Rhein, Deutschland; 2 Heidelberg, Deutschland

**Keywords:** Pflegegeleitete Demenzberatung, Dyadische Intervention, Ambulantes Setting, Krankheitsbewältigung, Multiperspektivische Evaluation, Nurse-led dementia counseling, Dyadic intervention, Outpatient setting, Coping, Multi-perspective evaluation

## Abstract

**Hintergrund und Fragestellung:**

Die Lebensqualität von Personen mit Demenz und ihren Angehörigen wird entscheidend von einer stabilen häuslichen Versorgungssituation beeinflusst. Bislang spielen dyadische Interventionskonzepte keine große Rolle. In dieser Studie wurde ein aufsuchendes dyadisches Unterstützungsprogramm für Personen mit Demenz und Angehörige erstmals durch Pflegeexpert:innen Demenz (PED) durchgeführt. Der lebensweltliche Nutzen eines dyadischen Ansatzes zur Alltags- und zur Krisenbewältigung im häuslichen Umfeld steht dabei im Fokus. Erweist sich das Programm als ein passendes Angebot für Dyaden in ländlichen Regionen? Und kann das ursprünglich für Therapeuten entwickelte Programm von PED durchgeführt werden?

**Methoden:**

Mit 12 Dyaden wurden episodische Interviews geführt, ergänzt durch Fokusgruppen (*n* = 5 Angehörige, *n* = 2 Pflegeexpert:innen). Die Interviews wurden aufgezeichnet, regelgeleitet transkribiert und qualitativ-inhaltsanalytisch ausgewertet.

**Ergebnisse:**

Vorgestellt werden Ergebnisse einer qualitativen Evaluation mit multiperspektivischem Ansatz. Der Benefit der Teilnehmenden ist abhängig von verschiedenen Aspekten (z. B. Dyadenbeziehung, Demenzschweregrad), der aufsuchende Charakter ist dabei zentral. Der dyadische Ansatz wird als hilfreich angesehen, es besteht jedoch zusätzlich ein Bedarf an Einzelgesprächen. Das Programm kann durch PED angewendet werden; der Professionswechsel kann von Vorteil sein. Fraglich ist, ob eine zeitlich limitierte Unterstützung über den gesamten Krankheitsverlauf trägt.

**Schlussfolgerungen:**

Die Lebenssituationen von Dyaden lassen sich durch das pflegegeleitete Programm verbessern. Die Möglichkeit von Einzelgesprächen sollte integriert und eine Folgebetreuung gegeben sein. Die Ergebnisse zeigen die Notwendigkeit aufsuchende Angebote in der Versorgung von Personen mit Demenz auszubauen, um Stress zu reduzieren und Identitäts- und Handlungsressourcen zu stärken.

## Abstract (en)

**Background and objective:**

The quality of life of persons with dementia and their relatives crucially depends on whether they succeed in establishing a stable home care situation. In this study, an outreach dyadic support program for persons with dementia and relatives was carried out by dementia nursing experts (DNE) for the first time.

The aim of the nurse-led dyadic support program focuses on the lifeworld benefits for dyads and the benefits towards coping with dementia and crisis management in their home environment. Does it turn out to be a suitable offer for dyads in rural areas? Could the program originally developed for therapists be carried out by DNE?

**Methods:**

Using an interview guideline, 12 episodic interviews were conducted with dyads and additionally two focus groups (*n* = 5 relatives and *n* = 2 dementia nursing experts). The interviews were recorded, transcribed verbatim and analyzed using content analysis.

**Results:**

The results of a qualitative evaluation with a multiperspective approach are presented. The participating dyadic benefit depends on several factors, e.g. dyadic relationship, stage of dementia, in different ways. The outreach approach is therefore important. The dyadic setting is mostly seen as helpful, a need for additional one-to-one meetings is desired. The program can be used by DNE, the shift can even be an advantage. It is questionable whether temporary support lasts over the entire course of the disease.

**Conclusion:**

The results show that home care situations can be improved for dyads through the nurse-led program, they can even benefit by DNE. The possibility of one-to-one meetings and follow-up care should be given. The outreach approach is crucial for rural areas. The results point to the need for more outreach care for persons with dementia in order to reduce stress and strengthen identity and action resources.

Mit einer Demenz so gut wie möglich zu Hause leben: Diese Herausforderung stellt sich der betroffenen Person und dem pflegenden Angehörigen gleichermaßen. Dabei macht es einen entscheidenden Unterschied, ob man sich der Aufgabe gemeinsam stellen kann, oder ob jeder für sich mit der Situation zurechtzukommen versucht. Psychoedukative Unterstützungsangebote haben diese dyadische Perspektive bisher kaum berücksichtigt. In der hier vorgestellten Studie wurde ein zugehendes und pflegegeleitetes Unterstützungsprogramm evaluiert, das ganz gezielt die gemeinsame Krankheitsbewältigung stärken soll.

## Hintergrund

Derzeit leben etwa 1,6 Mio. von einer Demenz betroffene Personen in Deutschland [1]. Ein großer Teil dieser Personen wird, meist durch Angehörige, zu Hause betreut. In der Häuslichkeit und insbesondere im ländlichen Raum stehen betroffene Personen und ihre Angehörigen vor zusätzlichen Herausforderungen bei der Inanspruchnahme von Angeboten, z. B. wegen fehlender Erreichbarkeit aufgrund der räumlichen Entfernung oder bestehenden Mobilitätseinschränkungen [12]. Auch sind pflegende Angehörige von Personen mit einer Demenz stark gesundheitlich belastet [7], v. a. wenn im Verlauf der Demenz herausforderndes Verhalten auftritt, welches häufig ein Grund für eine vollstationäre Unterbringung der Person mit Demenz darstellt.

Multimodale Angebote für pflegende Angehörige und Personen mit einer Demenz können die Institutionalisierung verzögern und zu einer Reduktion von herausforderndem Verhalten führen [9]. Bekannt ist auch, dass Personen mit einer Demenz und ihre Angehörigen Selbstmanagementfähigkeiten entwickeln und sich bemühen, vorhandene Ressourcen zu mobilisieren, die sie bei der Bewältigung ihrer Lebenssituation unterstützen können [14]. Es zeigt sich, dass Familien genau darin fachkundige und bedürfnisorientierte Beratung und Anleitung benötigen [13, 14]. Häusliche Beratung und Interventionen von Pflegeexpert:innen Demenz reduzieren herausfordernde Verhaltensweisen von zu Hause lebenden Personen mit Demenz signifikant [4]. Diese psychosozialen Unterstützungs- und Beratungsangebote leisten einen wichtigen Beitrag, wenn sie möglichst frühzeitig, passgenau und begleitend erfolgen [8, 12]. Zur Förderung der Lebensqualität ist es zudem bedeutsam, die individuellen Beziehungen zwischen den Personen mit einer Demenz und ihren pflegenden Angehörigen zu berücksichtigen [10]. Die gleichrangige Einbeziehung der Perspektiven von Hauptbetroffenen und Angehörigen stellt einen weiteren Schritt dar, um die gemeinsame und die geteilte Lebenswelt unter den Bedingungen der Demenz in Dyaden besser verstehen zu können.

Arbeiten über Stress-Coping-Prozesse in dyadischen Beziehungen verweisen klar auf die Notwendigkeit dyadischer Interventionen und auf das Potenzial, die gemeinsame Krankheitsbewältigung zu stärken [2]. Dennoch spielen dyadische Angebote bislang keine große Rolle; bestehende Interventionskonzepte richten sich in der Regel entweder an die Angehörigen oder an die Personen mit der Demenz [15, 16].

Im Rahmen eines vom Ministerium für Soziales, Arbeit, Gesundheit und Demografie Rheinland-Pfalz geförderten Modellprojektes wurde ein für die Anwendung durch Therapieberufe erprobtes dyadisches Therapiemanual [5] in leicht adaptierter Form durch Pflegeexpert:innen Demenz^1^ durchgeführt und dahingehend evaluiert, inwieweit es in der ambulanten gerontopsychiatrischen Beratung durch die neue Anwendergruppe erfolgreich eingesetzt werden kann. Um die ambulante Versorgungsrealität bei Demenz abzubilden, wurde der Dyadenbegriff weit gefasst, und sowohl Paardyaden als auch Eltern-Kind-Dyaden wurden einbezogen.

Den Hintergrund bildeten Erfahrungen aus der Arbeit der aufsuchenden gerontopsychiatrischen Fachambulanz, wo Pflegeexpert:innen Demenz (PED) in Beratung und Psychoedukation eine tragende Rolle übernehmen. Hieraus wurde die Annahme abgeleitet, dass PED nicht nur in der Lage sind, das dyadische Therapiemanual erfolgreich einzusetzen, sondern dass die pflegespezifische Expertise (u. a. Beziehungsarbeit, lebensweltorientierte Einschätzung der Situationsspezifika von Dyaden, hermeneutisches Fallverstehen) gegenüber der Ursprungsvariante sogar Vorteile bieten könnte. Diese Annahmen wurden im Rahmen eines „Mixed-methods“-Ansatzes evaluiert.

Das durchgeführte DYADEM-Programm basiert auf sozio- und verhaltenstherapeutischen Elementen und umfasst 9 Module, von denen 7 als Hausbesuche (im Umfang von jeweils ca. 50 min) und 2 als telefonische Beratungseinheiten stattfinden. Grundlegend in allen Modulen ist der in der psychiatrisch-therapeutischen Arbeit etablierte trialogische Ansatz: Das heißt, Beratende, Person mit der Demenz und Angehörige sind als Kommunikationspartner:innen gleichberechtigt. Thematische Schwerpunkte des Programms sind die Aufklärung und Beratung zur Demenzerkrankung, Vermittlung von Methoden zur Stressbewältigung, Kommunikations- und Problemlösetraining, Konflikt- und Krisenmanagement, Übungen zum Erkennen und zur verstärkten Nutzung alltagsbezogener und sozialer Ressourcen sowie Beratung zu Hilfs- und Unterstützungsangeboten [5].

Die hier vorgestellte qualitative Studie ist ein Teilprojekt der durchgeführten Evaluationsstudie. Die Ergebnisse der quantitativen Analysen können an anderer Stelle nachgelesen werden [17, 18].

## Ziel und Forschungsfragen

Ziel der qualitativen Studie war es, die Effektivität und Machbarkeit des pflegegeleiteten DYADEM-Programms aus Sicht aller Beteiligten – Personen mit der Demenz, Angehörige und Pflegeexpert:innen – zu evaluieren.

1. Wie wurde das durchgeführte Programm von den Akteur:innen wahrgenommen, welche Erfahrungen wurden dabei gemacht, und inwieweit wurde das Programm mit Blick auf die Alltags- und Krankheitsbewältigung als hilfreich erlebt?
2. Inwieweit ist das Programm von der ursprünglichen professionellen Verortung im therapeutischen Bereich in das Aufgabenfeld von Pflege übertragbar? Können Teilnehmende durch diese Übertragung sogar profitieren?

## Methodik

Unmittelbar nach Programmabschluss wurden episodische Interviews mit den Dyaden durchgeführt, weiterhin fand nach 6‑monatigem Abstand zum Erstinterview eine Fokusgruppe mit Angehörigen (*n* = 5) statt. Die beiden Pflegeexpert:innen wurden zu 2 Zeitpunkten (Mitte und Ende der Projektphase) gemeinsam interviewt.

Bei den Dyadeninterviews wurde ein gestuftes Vorgehen gewählt: Da, entsprechend dem dyadischen Programmansatz, die gemeinsame Perspektive auf die gemachten Erfahrungen erfasst werden sollte, ohne etwaige Unterschiede in der individuellen Perspektive zu vernachlässigen, wurde eine Kombination von Dyaden- und Einzelinterview gewählt. Inwieweit die Einbeziehung der Personen mit Demenz dabei gelingen würde, stellte von vornherein eine offene Frage dar: Einerseits gibt es viele Hinweise dafür, dass durch eine Anpassung der Interviewsituation selbst bei mittelgradiger Demenz noch Interviewforschung möglich ist [3]. Entsprechend wurde das Interview sprachlich (einfache Sprache) und methodisch (hermeneutisch ausgerichtete Gesprächstechnik) angepasst. Andererseits blieb gleichwohl die retrospektive Evaluationsperspektive problematisch: Inwieweit die Personen mit der Demenz sich an die nahe zurückliegenden Programmerfahrungen explizit erinnern könnten, konnte im Vorfeld nicht eingeschätzt werden. Als Erinnerungshilfe wurden Fotos der Pflegeexpert:innen eingesetzt.

## Stichprobe der Dyaden

Insgesamt wurden 12 Interviews (*n* = 10 Paardyaden und *n* = 2 Eltern-Kind-Dyaden) von Februar 2018 bis Januar 2019 geführt, wobei eine Person mit einer Demenz nicht teilnehmen konnte, da sie sich zum Zeitpunkt des Interviews in der Kurzzeitpflege befand. Die Anzahl entspricht gut einem Drittel der Gesamtstichprobe der in das Programm einbezogenen Dyaden.

Die Altersspanne lag zwischen 50 und 85 Jahren. Bis auf 2 Dyaden lebten alle Teilnehmenden im ländlichen bzw. im kleinstädtischen Wohnumfeld. Alle Interviews fanden bei den Dyaden zu Hause statt. Die Gesamtdauer der Interviews pro Dyade lag zwischen 52 und 108 min.

## Auswertung

Die Interviews wurden regelgeleitet verbatim transkribiert und nach der Methode der inhaltlich strukturierenden qualitativen Inhaltsanalyse [6], unterstützt durch die Analysesoftware MAXQDA, Version 18 (VERBI – Software. Consult. Sozialforschung. GmbH, Berlin, Deutschland), ausgewertet. Bis auf einen Fall gab es in der kommunikativen Verständigung mit den Personen mit einer Demenz kaum Einschränkungen. Die Interviewpartner:innen mit einer Demenz äußerten sich bis auf 2 Interviews jedoch kaum oder nur sehr vage zum Programm, gaben teilweise auch an, sich nicht mehr zu erinnern. Daher basieren die im Folgenden dargestellten Wahrnehmungen, wenn nicht explizit als Äußerung von Personen mit einer Demenz kenntlich gemacht, auf den Aussagen der Angehörigen.

Zur Auswertung der beiden Fokusgruppen (Angehörige und PED) wurde eine deduktive Strategie der qualitativen Inhaltsanalyse verwendet [6].

Durch die Wahl der qualitativen Inhaltsanalyse konnte das unterschiedliche Material mit einheitlicher Methodik ausgewertet werden. Die einzelnen Bereiche (Interviews sowie jede Fokusgruppe) wurden separat ausgewertet und werden im Ergebnisteil entsprechend dargestellt.

## Ergebnisse

Die dargestellten Ergebnisse umfassen 3 Schwerpunkte: (1) Beitrag des Programms zur Krankheitsbewältigung Angehörigenperspektive, (2) Erleben des triadischen Settings aus (hierzu können Aussagen beider Dyadenpartner:innen einbezogen werden), (3) Übertragbarkeit des Programms auf die Pflegeexpert:innen Demenz: Zu dieser Fragestellung werden die Ergebnisse der Fokusgruppen mit den beiden PED berichtet.

## Krankheitsbewältigung aus Angehörigenperspektive

Der aufsuchende Ansatz stellte einen bedeutsamen Aspekt für die Inanspruchnahme dar, und die Angehörigen erlebten dies als erleichternd, u. a. da Auswärtstermine der Person mit einer Demenz häufig schwer zu vermitteln waren. Zudem erlebten sie ihre/n Dyadenpartner:in mit Demenz in der häuslichen Umgebung selbstsicherer.

Im Hinblick auf den Nutzen des Programms für den Umgang mit der Erkrankung lassen sich 3 Dimensionen unterscheiden. Die *erkenntnisbezogene Dimension* beinhaltet einen von den Angehörigen wahrgenommenen Lernzuwachs. Dieser kann sich sowohl darauf beziehen, die Situation der Person mit der Demenz jetzt besser verstehen zu können, oder auf eine verbesserte Wahrnehmung von Alltagsaktivitäten, insbesondere was die Bewahrung positiver Aspekte angelangt:

„… und da hab ich so gemerkt (…) das ist auch wichtig, um im Kopf lebendig zu bleiben, und ist auch wichtig für unsere Gemeinsamkeit. Also, das war eigentlich mit so das wichtigste Ergebnis. Dass diese gemeinsamen Unternehmungen/dass die so wichtig genommen werden müssen, sodass sie in den Lebensalltag eingebaut werden müssen“ (D12_1, 193–195).

Die *emotionsbezogenen Wirkungen* zeigen sich in einer wahrgenommenen seelischen Entlastung und Festigung sowie in der Erkenntnis, dass eigene Herausforderungen und Belastungen von anderen Menschen geteilt werden. „Das tat mir sehr gut. Das war wie so ’ne nachträgliche Therapie auch für mich“ (D06_1, 84–85).

Die dritte Ebene umfasst *Verhaltensänderungen von Angehörigen*. „Da erinnere ich mich wirklich ganz oft dran, wo ich denke, nee, brauchst du nicht zu diskutieren. Es geht auch so“ (D05_1, 158–160). Oder die Akzeptanz, dass es in der Wohnung nicht mehr so ordentlich aussehen muss, wie man es von früher gewohnt war.

Als hilfreich wurden hier auch die gelernten Methoden zur Selbstsorge erlebt: „… dass ich wieder dran denke, an Entspannung für mich. Dass ich mir auch mal die Zeit für mich nehme“ (D3_1, 84–85). Beim *Zugang zu Hilfen* ging es nicht nur darum, um Unterstützungsressourcen und professionelle Hilfen zu wissen, sondern diese auch als hilfreich zu erkennen und offensiver zu nutzen: „… und dass ich auch mehr dran denke, auf welche Betreuungsangebote kann ich zurückgreifen und die auch nutze“ (D3_1, 85–86).

Nicht allen Angehörigen gelingt es, die Krankheit Demenz zu akzeptieren. Der Umgang mit bestimmten herausfordernden Verhaltensweisen, insbesondere mit aggressivem Verhalten der Person mit einer Demenz, wird auch weiterhin im Alltag als große Belastung erlebt. In den *zukunftsbezogenen Äußerungen* der Teilnehmenden finden sich sowohl Aspekte der Zuversicht und Hoffnung als auch der Unsicherheit und Sorge. Gefragt nach zukunftsbezogenen Bedarfen wird deutlich, dass die Zuversicht in künftige Hilfemöglichkeiten variiert, insbesondere bei Veränderungen des Gesundheitszustandes. „Im Moment ist ja eigentlich so weit alles in Ordnung. Das kann sich natürlich verschlechtern“ (D10_1, 130–131).

Insgesamt können alle Angehörigen Aspekte beschreiben, in denen die Teilnahme am Programm ihnen hilfreich war. Zugleich wird in den Äußerungen deutlich, dass die Beziehungsvorgeschichte, die aktuelle emotionale Verbundenheit sowie vorhandene Kommunikationsschemata die gemeinsame Krankheitsbewältigung und auch das Ausmaß des erlebten Nutzens des Programms beeinflussen.

## Erleben des triadischen Settings

Die Pflegeexpert:innen Demenz wurden als Vertrauenspersonen und als „Sparringspartner“ wahrgenommen. Positiv wurde die partizipative Arbeitsweise mit den Pflegeexpert:innen erlebt, die Qualität der Beziehung und das Gespräch auf Augenhöhe. Eine Person mit fortgeschrittener Demenz antwortet spontan beim Blick auf das Foto: „Der fehlt mir.“ Sowohl von Angehörigen als auch vonseiten einer Person mit der Demenz wird ein positiver Gegensatz zu Erfahrungen in Gesprächen mit Ärzt:innen beschrieben:

„… Bis dahin wars halt so, dass ich/dass wir immer/gut wir waren auch beim Doktor, beim Neurologen und so. Da ist das dann auch Thema, aber nochmal anders als beim [PED]. Da [mit dem/der PED] gings dann doch darum, wie mit dieser Erkrankung gut zu leben ist“ (D12_1, 185–190, Angehörige).

Das trialogische Setting kann den kommunikativen Raum der Dyade auch erweitern. Eine Angehörige: „Ich war manchmal erstaunt, wie (die PmD) über mich denkt (…), weil das hätt ich sonst nicht erfahren. Das hätt (er/sie) sonst nicht gesagt, wenn niemand da gewesen wär“ (D11_1, 171–172).

Angehörige beschreiben es wiederholt als entlastend, wenn Schwierigkeiten oder Konflikte für die Pflegeexpert:innen erkennbar werden, denen sie sich sonst alleine gegenübergestellt sehen. Obgleich die gemeinsame Krankheitsbewältigung im Vordergrund steht, nehmen einige Angehörigen ein ungleiches Kräfteverhältnis in der Kommunikation wahr, die welches in der trialogischen Kommunikation eine Herausforderung darstellt:

„(…) Weil ich denke, es gibt ja auch mal Sachen, die man gerne alleine besprechen möchte. Es kann sein, wenn ich dabei bin, dass [er/sie] sich gehemmt fühlt oder möchte oder kann nicht irgendwas sagen. Viel freier wahrscheinlich, wenn ich nicht dabei bin, und umgekehrt das Gleiche“ (D07_1, 827–834). Aus diesem Grund, und auch um selbst offener über bestimmte Probleme reden zu können, sprechen sich mehrere Angehörige dafür aus, dass es neben den gemeinsamen Sitzungen auch Einzelgespräche geben sollte.

## Übertagbarkeit des Programms auf die Pflegeexpert:innen Demenz

Beide Pflegeexpert:innen beschreiben die Arbeit mit dem Programm als anspruchsvoll, aber mit dem eigenen Qualifikations- und Erfahrungshintergrund als gut leistbar.

Die individuellen Lebenssituationen von Dyaden sind komplex und divers. Häufig ist die Ausgangssituation durch Überlastung, Überforderung und damit einhergehende Konflikt- und Krisensituationen gekennzeichnet. Eine Schlüsselrolle spielt die Fähigkeit des PED, spontan, flexibel und in lösungsorientierter Weise zu reagieren sowie achtsam mit Nähe und Distanz umzugehen.

Voraussetzungen zur Durchführung des Programms sind aus Sicht der PED hohe Empathie‑, Interaktions- sowie krankheits- und pflegespezifische Kompetenzen. Dabei seien die dem professionellen Pflegehandeln inhärente Lebensweltorientierung, pflegefachliche Wahrnehmungskompetenzen für Situationseinschätzungen, Ressourcenorientierung sowie eine beziehungsorientierte Arbeitsweise für das Arbeitsbündnis mit den Dyaden und das Gestalten eines geschützten Rahmens wesentlich.

Die PED beschreiben weiterhin, dass sie im triadischen Setting verschiedene Rollen einnehmen, z. B. als „Katalysator“, in dem sie Raum zum „Dampf ablassen“ geben, oder als Vertrauensperson, die die Dyaden motiviert, belastende Gefühle offen auszusprechen. In der Funktion des „Geburtshelfers für lösungsorientierte Gedanken“ werden bedeutsame Aspekte zur gemeinsamen Krankheitsbewältigung, die bei den Dyadenpartner:innen schon vorhanden sind, verbalisiert und damit bearbeitbar. Gleichzeitig sehen die Pflegeexpert:innen Demenz auch Handlungsgrenzen: Bestimmte Konflikte und Rollenkonstellationen können nicht aufgelöst werden und bedürfen weitergehender Interventionen.

## Diskussion

Insgesamt belegen die Ergebnisse eine hohe Akzeptanz für das Interventionsportfolio bei den teilnehmenden Dyaden. Das durch Pflegeexpert:innen Demenz durchgeführte dyadische Programm kann die gemeinsame Alltags- und Krankheitsbewältigung unterstützen. Eine Stabilisierung der Lebenssituation erleben die Dyaden durch spezifische Programminhalte (u. a. Ressourcenorientierung, Alltagsaktivitäten) sowie unspezifische Erkenntnisse wie der, nicht allein in dieser Situation zu sein.

Das trialogische Setting eröffnet einerseits Kommunikationsräume und zeigt andererseits ein „Kräfteungleichgewicht“ zwischen pflegenden Angehörigen und den Personen mit der Demenz auf, z. B. wenn es darum geht, sensible Themen zu adressieren. Die Möglichkeit von Einzelgesprächen sollte zukünftig mitbedacht werden [11].

Der Beziehungsaufbau und die individuelle, situative Fallbegleitung der Dyaden durch Pflegeexpert:innen Demenz stärken das Pflegearrangement, indem umfassende und passgenaue Gesprächs- und Unterstützungsleistungen lebensweltorientiert und partizipativ in einem geschützten Rahmen gestaltet werden. Pflegeexpert:innen Demenz verfügen über diese Kommunikationskompetenz und Fachexpertise im Kontext der Beziehungsarbeit mit Dyaden.

Es ist bekannt, dass neben dem aufsuchenden Charakter und der wahrgenommenen Lotsenfunktion [12] für die Inanspruchnahme von Beratungs- und Entlastungsleistungen die Vertrauensbasis, die Wertschätzung für pflegende Angehörige von Personen mit einer Demenz und Fachkompetenzen [11] förderlich sind. Eine demenzspezifische Wissenserweiterung für die Dyaden und Informationen zu Angebotsstrukturen sind zentral, um die häusliche Versorgungssituation zu stabilisieren und die Herausforderungen im Alltag besser bewältigen zu können.

Die größte Limitation des multiperspektivischen Designs lag darin, dass bei der Evaluation die Perspektive der Person mit einer Demenz nur unzureichend erfasst werden konnte. In dieser Studie war eine Erweiterung des retrospektiven Befragungsansatzes nicht leistbar. Für ähnliche Evaluationsprojekte sollten jedoch Alternativen wie beispielsweise der Einsatz von Mikroevaluationen in unmittelbarer zeitlicher Nähe zu einzelnen Interventionsmodulen erwogen werden.

## Schlussfolgerungen

In der qualitativen Evaluation ging es darum, Effektivität und Machbarkeit des pflegegeleiteten Programms aus Sicht der verschiedenen Beteiligten – Personen mit einer Demenz, Angehörige und Pflegeexpert:innen – zu ermitteln. Die Lebenssituationen bei vorliegender Demenz lassen sich, zumindest aus Angehörigenperspektive, durch einen dyadischen Ansatz verbessern. Fraglich ist, ob eine zeitlich limitierte Unterstützung über den gesamten Krankheitsverlauf trägt. Die Möglichkeit der Folgebetreuung sollte gegeben sein. Das Programm kann durch Pflegeexpert:innen Demenz angewendet werden, und es gibt Hinweise darauf, dass es vom Professionswechsel profitieren kann. Der aufsuchende Ansatz ist für den ländlichen Raum entscheidend. Über den Fokus der Studie hinaus verweisen die Ergebnisse auf die Notwendigkeit, über eine mehr aufsuchende Versorgung von Dyaden nachzudenken, um Stress zu reduzieren und Identitäts- und Handlungsressourcen zu stärken.

## Fazit für die Praxis

Ein aufsuchendes dyadisches Unterstützungsprogramm, das von Pflegeexpert:innen Demenz durchgeführt wird, leistet einen wichtigen Beitrag zur dyadischen Krankheits- und Alltagsbewältigung bei Demenz in ländlichen Regionen. Eine bedarfsorientierte längerfristige Begleitung der Dyaden ist wünschenswert, um die häusliche Situation nachhaltig zu stabilisieren.

## Footnotes

1 Die Pflegeexpert:innen Demenz sind Pflegefachpersonen mit spezifischer Fachqualifikation Gerontopsychiatrie und langjähriger Berufserfahrung.

## References

[1] Deutsche Alzheimer Gesellschaft e. V. (DAlzG) (2020) Die Häufigkeit von Demenzerkrankungen. Informationsblatt 1. https://www.deutsche-alzheimer.de/fileadmin/alz/pdf/factsheets/infoblatt1_haeufigkeit_demenzerkrankungen_dalzg.pdf. Zugegriffen: 25. Nov. 2022

[2] Bodenmann G (1997). Dyadisches Coping: theoretischer und empirischer Stand. Z Familienforsch.

[3] Bödecker F, Schneider A, Köttig M, Molnar D (2015). Wie forschen mit Menschen mit Demenz? Probleme, Lösungen und Fragen. Forschung in der Sozialen Arbeit: Grundlagen – Konzepte – Perspektiven.

[4] Callahan CM, Boustani MA, Unverzagt FW, Austrom MG, Damush TM, Perkins AJ, Hendrie HC (2006). Effectiveness of collaborative care for older adults with Alzheimer disease in primary care: a randomized controlled trial. JAMA.

[5] Häusler A, Krause-Köhler K, Niemann-Mirmehdi M, Nordheim J, Rapp M (2014). Psychosoziale Therapie bei beginnender Demenz. Das DYADEM-Unterstützungsprogramm für Menschen mit Demenz und ihre Partner.

[6] Kuckartz U (2018). Qualitative Inhaltsanalyse. Methoden, Praxis, Computerunterstützung.

[7] Kurz A, Wilz G (2011). Die Belastung pflegender Angehöriger bei Demenz. Entstehungsbedingungen und Interventionsmöglichkeiten. Nervenarzt.

[8] Nordheim J, Rapp M (2016) Unterstützungsprogramm für Menschen mit Demenz sowie ihre pflegenden Angehörigen. Durchführung und Evaluation eines Trainings- und Unterstützungsprogramm für Patient-Angehörigen-Dyaden bei leichter bis mittelschwerer Demenz im ländlichen Versorgungsgebiet. htpp://www.zqp.de/wp-content/uploads/Abschlussbericht_DYADEM.pdf. Zugegriffen: 1. März 2021

[9] Mantovan F, Ausserhofer D, Huber M, Schulc E, Them C (2015). Interventionen und deren Effekte auf pflegende Angehörige von Menschen mit Demenz – Eine systematische Literaturübersicht. Pflege.

[10] Niemann-Mirmehdi M, Soellner R, Kalus P, Richert A, Heinz A, Rapp M (2008). Psychosoziale „TANDEMGRUPPEN“ . Evaluation sozialtherapeutischer Tandemgruppen zur Frühförderung der Krankheits- und Alltagsbewältigung demenziell erkrankter Patienten und ihrer Angehörigen. Z Gerontopsychol Psychiatr.

[11] Prick AE, de Lange J, van’t Leven N, Pot AM (2014). Process evaluation of a multicomponent dyadic intervention study with exercise and support for people with dementia and their family caregivers. Trials.

[12] Reichert M, Hampel S, Reuter V (2016). Mobile Demenzberatung als niedrigschwelliges Hilfeangebot für pflegende Angehörige. Z Gerontol Geriat.

[13] Smits CHM, de Lange J, Dröes RM, Meiland F, Vernooij-Dassen M, Pot AM (2007). Effects of combined intervention programmes for people with dementia living at home and their caregivers: A systematic review. Int J Geriat Psychiatry.

[14] Toms GR, Quinn C, Anderson DE, Clare L (2015). Help yourself: Perspectives on self-management from people with dementia and their caregivers. Qual Health Res.

[15] Van’t Leven N, Prick A-EJC, Groenewoud JG, Roelofs PDDM, de Lange J, Pot AM (2013). Dyadic interventions for community-dwelling people with dementia and their family caregivers: a systematic review. Int Psychogeriatr.

[16] Wadham O, Simpson J, Rust J, Murraya C (2016). Couples’ shared experiences of dementia: A meta-synthesis of the impact upon relationships and couplehood. Aging Ment Health.

[17] Wuttke-Linnemann A, Baake R, Fellgiebel A (2019). Dyadic wind of change: new approaches to improve biopsychological stress regulation in patients with dementia and their spousal caregivers. J Alzheimers Dis.

[18] Wuttke-Linnemann A, Henrici CB, Müller N, Lieb K, Fellgiebel A (2020). Bouncing back from the burden of dementia: Predictors of resilience from the perspective of the patient, the spousal caregiver, and the dyad—An exploratory study. GeroPsych.

